# Increased Susceptibility for Superinfection with *Streptococcus pneumoniae* during Influenza Virus Infection Is Not Caused by TLR7-Mediated Lymphopenia

**DOI:** 10.1371/journal.pone.0004840

**Published:** 2009-03-17

**Authors:** Sabine Stegemann, Sofia Dahlberg, Andrea Kröger, Marcus Gereke, Dunja Bruder, Birgitta Henriques-Normark, Matthias Gunzer

**Affiliations:** 1 Otto-von-Guericke University, Institute of Molecular and Clinical Immunology, Magdeburg, Germany; 2 Helmholtz Center for Infection Research, Research group Immuneregulation, Braunschweig, Germany; 3 Department of Microbiology, Tumor and Cell Biology, Karolinska Institutet & the Swedish Institute for Infectious Disease Control, Stockholm, Sweden; 4 Helmholtz Center for Infection Research, Department of Gene Regulation and Differentiation, Braunschweig, Germany; Institut Pasteur, France

## Abstract

Influenza A virus (IAV) causes respiratory tract infections leading to recurring epidemics with high rates of morbidity and mortality. In the past century IAV induced several world-wide pandemics, the most aggressive occurring in 1918 with a death toll of 20–50 million cases. However, infection with IAV alone is rarely fatal. Instead, death associated with IAV is usually mediated by superinfection with bacteria, mainly *Streptococcus pneumoniae*. The reasons for this increased susceptibility to bacterial superinfection have not been fully elucidated. We previously demonstrated that triggering of TLR7 causes immune incompetence in mice by induction of lymphopenia. IAV is recognized by TLR7 and infections can lead to lymphopenia. Since lymphocytes are critical to protect from *S. pneumoniae* it has long been speculated that IAV-induced lymphopenia might mediate increased susceptibility to superinfection. Here we show that sub-lethal pre-infections of mice with IAV-PR8/A/34 strongly increased their mortality in non-lethal SP infections, surprisingly despite the absence of detectable lymphopenia. In contrast to SP-infection alone co-infected animals were unable to control the exponential growth of SP. However, lymphopenia forced by TLR7-triggering or antibody-mediated neutropenia did not increase SP-susceptibility or compromise the ability to control SP growth. Thus, the immune-incompetence caused by transient lympho- or leukopenia is not sufficient to inhibit potent antibacterial responses of the host and mechanisms distinct from leukodepletion must account for increased bacterial superinfection during viral defence.

## Introduction

Influenza A virus (IAV) belongs to the class of orthomyxoviridae [1], [2] and presents with high genetic variability which is the cause for regularly occurring epidemics [2] or world-wide pandemics [3]. The 20th century has seen three IAV-pandemics, the most aggressive one being the “Spanish Flu” of 1918/1919. The 1918 IAV-variant [4], [5] rapidly spread over the globe reaching the most remote places such as Spitzbergen or Alaska causing 20–50 million deaths world-wide [3], [6].

The major reason for this large mortality was not the IAV-infection per se but rather secondary bacterial superinfections, often caused by *Streptococcus pneumoniae* [7], [8]. Supporting this notion, vaccination against *S. pneumoniae* can prevent 31% of IAV-associated pneumonias [9]. Thus, there seems to be a particularly lethal synergism between IAV and *S. pneumoniae* [7], [10]. *S. pneumoniae* is a Gram-positive, encapsulated, facultatively anaerobic bacterium [11] that is considered the most common bacterial respiratory tract pathogen. It causes otitis media and sinusitis, but is also a major contributor to community acquired pneumonia with mortality rates as high as 20% in patients with concurrent septicaemia [11]–[13]. The natural host-defence comprises complement-mediated phagocytosis and killing by polymorphonuclear neutrophil granulocytes (PMN). Serotype-specific antibodies of the host aid in this process and form the basis for preventive vaccination [9], [14]. Recently also CD4+ T cells have been implicated in the early control of the infection [11], [15], [16].

Although IAV-mediated predisposition for bacterial superinfections was initially observed almost 200 years ago [7] the molecular and cellular mechanisms for this lethal synergism are still not fully elucidated. A number of explanations exist (comprehensively reviewed in [7]). The most widely used concept is focussed on the destruction of the respiratory epithelium by IAV allowing increased adhesion of bacteria to the tracheal wall and thus better retention and growth of pneumococci [10]. However, also less destructive variants of IAV are able to induce lethal synergism in a mouse model [10] arguing for additional mechanisms. E.g. a massive induction of pro-inflammatory cytokines was observed in IAV-infected animals recruiting large numbers of PMN which ultimately destroy the lung tissue [7], [17]. However, other groups could demonstrate the opposite, a strongly reduced recruitment of PMN during pneumococcal infection in mice 4–6 weeks after recovering from IAV [18]. This was associated with a decreased response of alveolar macrophages (AM) in IAV-infected mice in response to Toll-like receptor (TLR) ligands of bacterial origin leading to their inability to produce neutrophil attracting chemokines such as Mip-2 and KC [18]. The latter study, however, fails to explain why a natural mechanism should exist that renders animals highly susceptible to secondary bacterial infections for several weeks after a single viral infection. It is difficult to imagine how such a process should have survived through evolution in the presence of a constant bacterial threat.

It seems more conceivable that immediate mechanisms directly associated with the viral defence process might be responsible for the increased susceptibility to bacterial superinfection during ongoing antiviral action and not after successful viral depletion. Along those lines it has been demonstrated, that impairment in AM phagocytosis of pneumococci 8–9 days after IAV infection was dependent on the reduction of the scavenger receptor MARCO on AM via T cell-derived interferon γ [19]. MARCO is one of the major receptors responsible for the uptake of *S. pneumoniae* by AM [15].

While massive production of T cell-derived interferon γ is clearly a hallmark of antiviral immune responses, also another change in virally infected hosts is frequently observed, which is leukopenia mediated by type 1 interferons [20]–[22]. Indeed it has been shown that increased susceptibility of mice to superinfection with *Listeria monocytogenes* after LCMV infection is caused by enhanced apoptosis of PMN leading to leukopenia and drastically reduced PMN infiltration at bacterial infection sites [22].

Is it possible, that similar mechanisms underlie the increased pneumococcal superinfection of IAV infected hosts? The host response to IAV is initiated by TLR7- and RIG-I-mediated recognition of the viral genome [23]–[25] leading to a rapid and massive production of type I interferons which are able to establish a so-called antiviral state. We have shown that triggering of TLR7 by the specific ligand R-848 rapidly induces lymphopenia in mice lasting 36–48 hours [26]. This lymphopenia renders animals unable to mount peripheral immune responses [26]. Also IAV infections can cause massive lymphopenia [20] peaking at day 7 of the infection. In IAV and *S. pneumoniae* co-infection models the time point of highest susceptibility for the bacterial superinfection was at day 7 after IAV pre-infection [10], correlating precisely with the peak of lymphopenia seen during IAV infection [20]. Thus we reasoned that TLR7 induced lymphopenia might increase susceptibility to pneumococcal superinfection.

To test this we established a mouse model for co-infection of the mouse adapted IAV-strain PR8/A/34 [10] and the invasive *S. pneumoniae* strain TIGR4 [27], [28]. Individually, both infections were sublethal but showed strong synergistic action when combined. Here we demonstrate that peripheral lymphocyte-counts were not diminished in this model and also that forced depletion of lymphocytes or PMN did not render mice susceptible to superinfection with *S. pneumoniae*.

## Methods

### Mice

Female C57Bl/6 mice were purchased from Charles River (Sulzfeld, Germany) or Harlan Winkelmann (Borchen, Germany) at the age of 6 to 8 weeks. Mice were housed under specific pathogen-free conditions according to the guidelines of the regional animal care committee. All experiments were approved by the local ethical committees (for the HZI: Niedersächsisches Landesamt für Verbraucherschutz und Lebensmittelsicherheit, file number 33.42502-006/07 and for the KI: all the experiments were conducted in conformity with the European Communities Council Directive 86/609/EEC and the Swedish animal protection legislation.)

### Viral and bacterial pathogens

Influenza A virus PR8/A/34 (H1N1) [10] was grown on Madin-Darby canine kidney (MDCK) cells. Shortly, cells were infected with the virus and incubated at 35°C and 5% CO_2_ for 24 hours. Supernatant was harvested, spun down to remove cellular debris and used for mouse infection experiments. Supernatant from uninfected cells was obtained likewise and served as inoculum for uninfected control animals. For bacterial challenges *Streptococcus pneumoniae* TIGR4, an encapsulated strain of serotype 4 (ATCC BAA-334 [27], [28]), was grown overnight on blood agar plates (BD Diagnostic Systems, Columbia Agar with 5% sheep blood) from frozen stocks at 37°C and 5% CO_2_. Colonies were briefly inoculated into pre-warmed DS (dextrose- serum) medium (OXOID manual 1990), and then inoculated into pre- warmed C+Y (casamino acid & yeast extract [29]) medium, grown to midlogarithmic phase (OD_620_ = 0,5) and subsequently diluted in C+Y medium in order to obtain the appropriate concentrations for the mouse infections. Bacterial medium was produced by the Karolinska Microbiology Laboratory (Solna, Sweden).

### R-848 treatment

For systemic TLR7 triggering, R-848 (Axxora, Loerrach/Germany) was administered intraperitoneally in a volume of 100 µl PBS at a concentration of 25 µg/ml, resulting in a dose of 2,5 µg/mouse as described [26].

### RB6-8C5 antibody treatment

For PMN depletion experiments 100 µg of the monoclonal antibody RB6-8C5 recognizing mouse Ly-6G (Gr-1, BioXCell, West Lebanon/USA) was intraperitoneally injected in a volume of 100 µl PBS 24 h prior to *S. pneumoniae* infection. Control animals were likewise treated with 100 µg of control rat IgG (Sigma-Aldrich). To confirm PNM depletion, at different time points after injection, 30 µl blood were taken from the tail vein, the sample depleted of erythrocytes as described below, cells stained with Phyco-erythrin labelled RB6-8C5 and analysed using a BD FACSCalibur or FACSCanto flow cytometer and the Dako Summit software.

### Viral and bacterial infections

For viral and bacterial challenge, 7 to 9 week old mice were lightly anaesthetized by isofluorane inhalation. Holding the animals upright, the viral or bacterial inoculum was given onto the nostrils to be taken up by the mouse upon breathing.

For the MDCK-cell derived IAV PR8/A/34 virus stock the dose lethal to 50% of inoculated C57Bl/6 mice (LD_50_) was determined by the method of Reed and Muench [30]. Briefly, groups of mice were intranasally infected with 25 µl of appropriate dilutions of the virus stock in control medium. Body weight and health status were monitored, survival assessed over 14 days and the MLD_50_ determined by endpoint calculation. Mice having lost more than 25% of body weight were sacrificed and the infection considered lethal. For all following experimental mouse infections a dose of 0.04 MLD_50_ was chosen.

For *S. pneumoniae* TIGR4 challenge, bacteria were diluted in C+Y medium to a concentration of ∼5×10^6^ CFU/ml, verified by plating out 10-fold dilutions onto blood-agar plates. Mice were inoculated intranasally with 20 µl of bacterial solution (controls with medium alone), health status was monitored at least twice/day for seven days or until earlier sacrificing. Blood was analyzed for bacterial counts at 24, 48 and 72 h after infection. When observing impairment in health conditions animals were sacrificed and the infection regarded as lethal.

### Assessment of *S. pneumoniae* CFU counts in blood, lungs, tracheal and bronchoalveolar lavage

For the assessment of bacterial counts in blood, 5 µl of blood were taken from the tail vein, serially diluted in PBS and dilutions were plated onto blood agar.

For CFU counts in lung tissue, lungs of sacrificed mice were aseptically removed, collected in 1 ml of PBS, homogenized through a 100 µm cell strainer and serial dilutions in PBS plated on blood plates.

Nasopharyngeal lavage was obtained post mortem by flushing the nasopharynx through trachea and nares with PBS through a 20G canule inserted into the trachea. 100 µl of fluid were collected from the nares and used for plating serial dilutions on blood plates.

Bronchoalveolar lavage was collected by flushing the lungs once with 1 ml of PBS. To determine CFU counts the sample was serially diluted in PBS and plated on blood plates. CFUs on blood plates were counted after 16 h of incubation at 37°C/5% CO_2_.

### Quantification of peripheral blood lymphocytes

For the quantification of peripheral blood lymphocytes from either R-848 treated or IAV infected mice, the animals were bled by tail vein puncture. Blood samples of defined volume (30 µl blood) were obtained before, 1 hour, 24 hours and 48 hours after R-848 injection or once/day at the indicated time points post IAV infection. Blood samples were depleted of erythrocytes by osmotic shock through addition of red blood cell lysis buffer (0.15 M NH_4_Cl, 0.01 M KHCO_3_, 0.1 mM EDTA, pH7.2) and subsequent centrifugation to pellet lymphocytes. Cells were then stained for CD4^+^, CD8a^+^ and CD45R/B220^+^ (antibodies from BD Pharmingen, clone RM 4-5, 53-6.7 and RA3-6B2, respectively). Cell counts were acquired on BD FACSCanto or FACSCalibur flow cytometers. Acquisition of a defined volume was performed by analysing samples at a constant flow rate over a defined period of time. This allowed tracking of cell counts over various time points, expressing cell numbers as percentage of the starting value at time point 0. Data was analysed using the Dako Summit software.

### Analysis of lymphocyte subsets in lung tissue and bronchoalveolar lavage

Lungs were perfused with PBS, excised and finely minced on ice, followed by enzymatic digestion for 45 minutes at 37°C in Iscove's modified Dulbecco's medium (IMDM) containing 0,2 mg/ml Collagenase D (Roche), 10 µg/ml DNase (Sigma) and 5% fetal calf serum. After addition of EDTA (5 mM final concentration), suspensions were pelleted by centrifugation and depleted of erythrocytes by osmotic shock. Cells from BAL fluid were prepared by flushing the lung once with 1 ml PBS and centrifugation of the sample at 420×g for 10 minutes. For FACS analysis, Fc-block was performed through incubation with anti-mouse CD16/CD36 antibody (BD Pharmingen) followed by staining for mouse CD4, CD8, CD19, CD11b and Gr-1 (clones RM4-5, 53-6.7, 1D3, M1/70 and RB6-8C5, respectively). Data were acquired on a BD FACS Canto flow cytometer and analysed using DAKO Summit software.

### Statistical analysis

All statistical analyses shown were performed by paired, two-tailed *t* test and survival data compared by Kaplan Meier analysis log rank test using Graph Pad Prism software (Graph Pad Software, La Jolla/USA).

## Results

### Characterization of an IAV/*S. pneumoniae* co-infection model

**Figure 1:**
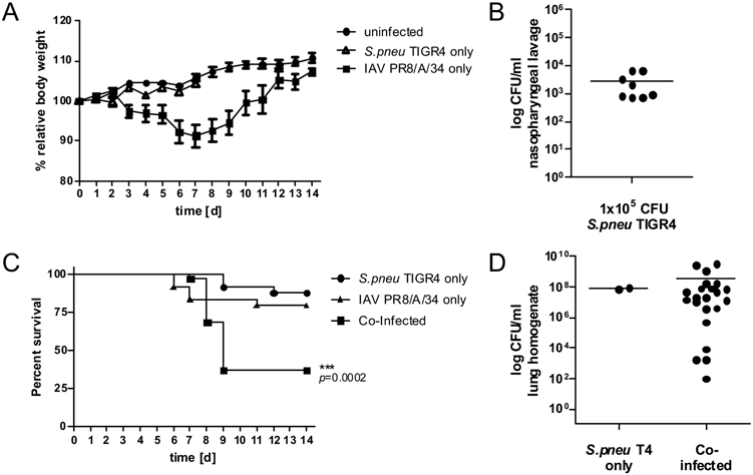
Influenza A virus infection predisposes for invasive disease through *Streptococcus pneumoniae*. (A) C57Bl/6 mice were intranasally inoculated with medium, 0.04 MLD_50_ Influenza A virus (IAV) PR8/A/34 or 1×10^5^ CFU *Streptococcus pneumoniae* TIGR4 (T4) and weighed daily. Body weight is shown as % relative to the starting weight. (B) *S. pneumoniae* CFU counts in tracheal lavage of survivors seven days after i.n. infection. (C) Survival rates of C57Bl/6 mice after i.n. infection with IAV PR8/A/34 alone (day 0), *S. pneumoniae* T4 alone (day 7) or *S. pneumoniae* T4 (day 7) following IAV (day 0). (D) CFU counts in lung homogenates of *S. pneumoniae* only infected and Influenza A virus pre-infected C57Bl/6 mice in which infection was lethal. All data shown are compiled from at least two independent experiments with groups of 5 or more mice. No bacteria could be detected in the lungs of mice surviving the infections (not shown).

To establish a model system for IAV/*S. pneumoniae* synergism in the mouse we established two sublethal infections with either the mouse-adapted viral strain PR8/A/34 or the pneumococcal strain TIGR4. 0.04 MLD_50_ of IAV caused a mild disease with a transient mean loss of max. 10% body weight up to day 7 of the infection, which was resolved by day 12 post infection (Fig. 1A). Likewise, a sublethal course of *S. pneumoniae* was established after infection with 1×10^5^ CFU that was not detectable by weight changes of infected animals (Fig. 1A). A productive infection was verified from nasopharyngeal lavages at day 7 p.i. in all infected animals (Fig. 1B). Survival-curves showed that both infections were sublethal to 80% (IAV) or 88% (*S. pneumoniae*) of all animals although single individuals could still succumb to the infection as seen elsewhere [10]. The combined sublethal infections with IAV followed by *S. pneumoniae* 7 days later were highly synergistic leading to 63% mortality within 2 days after the bacterial superinfection (Fig. 1C). The lung homogenates of animals succumbing to the co-infection showed high CFU in their tissues similar to the levels observed in the rare cases of lethal courses from single infections (Fig. 1D).

**Figure 2:**
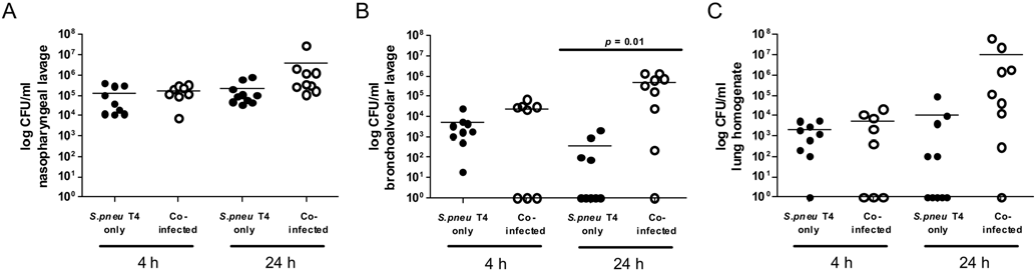
Influenza A virus infection renders mice unable to control *Streptococcus pneumoniae* growth in the upper and lower respiratory tract. C57Bl/6 mice were inoculated with medium (•) or 0.04 MLD_50_ Influenza A virus (IAV) PR8/A/34 (○) seven days before infection with 1×10^5^ CFU *Streptococcus pneumoniae* TIGR4 (T4). Mice were sacrificed 4 and 24 hours after bacterial infection and *S. pneumoniae* CFU counts in nasopharyngeal lavage (A), bronchoalveolar lavage (B) and lung homogenates (C) were assessed. Data show values of individual mice together with group means (horizontal lines) and are compiled from two independent experiments.

This suggested the inability to control the bacterial spread as cause for a lethal *S. pneumoniae* superinfection. Thus, we measured the extent of colonization at defined time points (4 and 24 h) after the bacterial infection either alone or in the co-infection model (Fig. 2). At 4 h p.i. animals in both models could still control bacterial spread to the same degree. However, at 24 h singly infected mice started to clear the bacterial infection in both the bronchoalveolar lavage (BAL) and lung-tissues, while co-infected mice failed to control bacterial proliferation at all sites tested (Fig. 2). These findings further supported that a defect in controlling bacterial growth occurred already early in superinfected mice.

### Absence of leukopenia in IAV/*S. pneumoniae* co-infected animals

**Figure 3:**
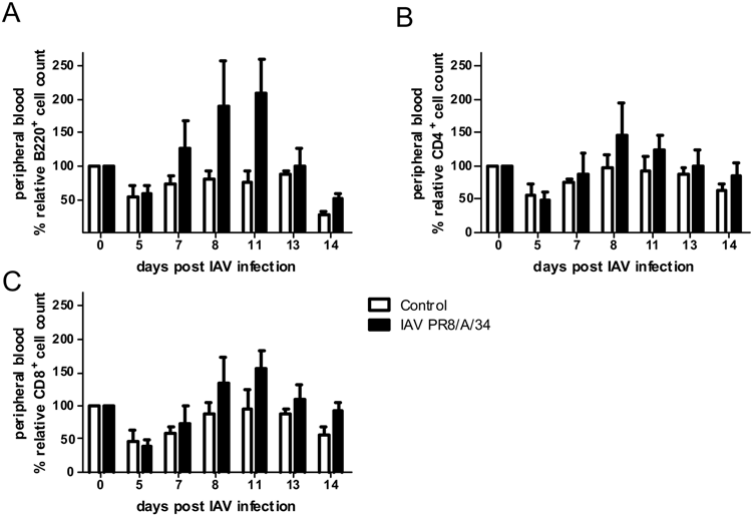
Analysis of peripheral blood lymphocyte counts shows no lymphopenia in the course of Influenza A virus infection. C57Bl/6 mice were intranasally inoculated with 0.04 MLD_50_ of Influenza A virus (IAV PR8/A/34) or medium (control) and repetitive blood samples on all animals were taken on different days p.i. The numbers of B220- (A), CD4- (B) and CD8-positive (C) cells in peripheral blood as determined by flow cytometry are shown as % relative to pre-infection levels. Data show representative results as means±s.e.m. of one out of two experiments with five mice per group.

We next asked whether a reduction of peripheral white blood cells (leukopenia) was associated with the course of the viral infection, as had been demonstrated elsewhere [20]. Thus, we measured the levels of peripheral blood B cells as well as CD4 and CD8 T cells at different time points after a sublethal IAV-infection. However, we never observed lymphocyte-numbers in IAV-infected animals that were below the levels found in mock-infected controls (Fig. 3). In fact, from day 7 up to 14 p.i. the lymphocyte-numbers in IAV-infected animals were higher as compared to controls (Fig. 3).

Thus, in the IAV-infection used here no detectable lymphopenia was evident in the animals despite a prominent viral-bacterial synergism. Therefore, the synergism observed here must be based on mechanisms distinct from peripheral lymphocyte-depletion.

### Forced lympho- or neutropenia does not lead to enhanced susceptibility to bacterial superinfection

**Figure 4:**
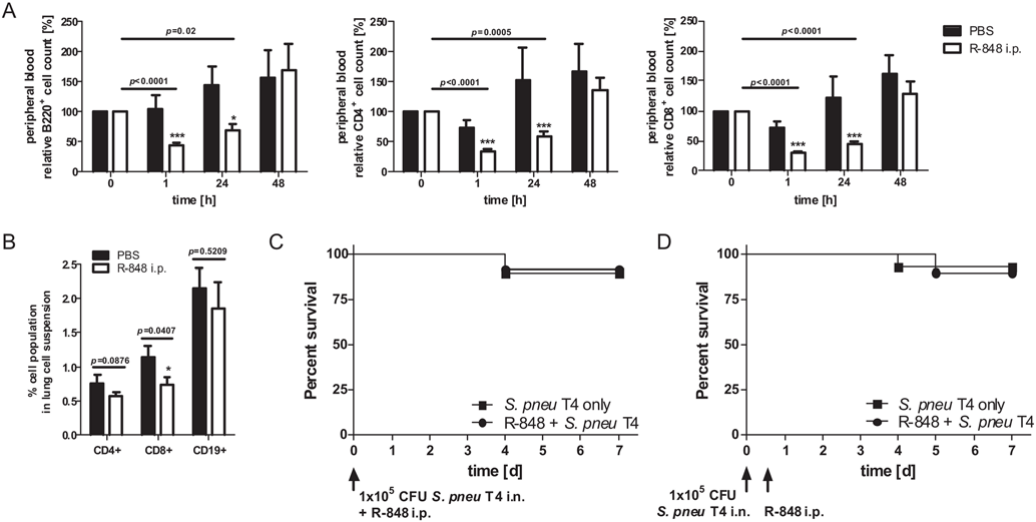
Systemic TLR7 triggering leads to transient peripheral blood lymphopenia but does not cause increased susceptibility for lethal *Streptococcus pneumoniae* infection in C57Bl/6 mice. (A) Mice were injected with PBS or R-848 intraperitoneally and bled from the tail vein immediately before, 1, 24 and 48 h after treatment. Samples were analysed for B220-, CD4- and CD8-positive cells by flow cytometry. Cell numbers are expressed as relative % compared to pre-treatment levels. Data show means±s.e.m. compiled from three independent experiments. (B) Likewise, lung tissue of R-848- or PBS-treated animals was analysed for the content of CD4^+^, CD8^+^ and CD19^+^ cells 1 h after treatment. Data show means±s.e.m. compiled from five individual animals. Additionally, different groups of mice were intranasally inoculated with 1×10^5^ CFU *Streptococcus pneumoniae* TIGR4 (T4) together with (C) or 12 hours before (D) intraperitoneal R-848 injection and survival was assessed. The lymphopenia in these mice was also verified (not shown). Data show compiled results of two independent experiments performed with five mice per group (*** p<0.001, * p<0.05).

Can leukopenia predispose for *S. pneumoniae* superinfection at all? In animal models and human patients virus-induced leukopenia coincides with increased susceptibility to bacterial superinfections [7], [22]. In addition, we have previously shown that TLR7-triggering with the imidazoquinoline R-848 induces lymphopenia and leads to transient immune-incompetence in the host [26]. Thus, using R-848 as trigger we tested whether forced lymphopenia would render animals hyper-susceptible to *S. pneumoniae*-infection. As shown for BALB/c mice [26], R-848 also induced profound lymphopenia in the C57Bl/6 mice used here (Fig. 4A). Like in BALB/c mice [26] this lymphopenia was evident at 1 h after application of R-848 and lasted for at least 24 h, being more pronounced in the T cell compartment as compared to the B cell pool (Fig. 4A). To verify, that R-848 treatment also affected lymphocyte numbers at the site of infection, we analyzed lung tissue for the content of CD4^+^, CD8^+^ and CD19^+^ cells. Indeed, although these cells only constituted a minor fraction in the analyzed lung tissues, all populations were further reduced by R-848 treatment (24%, 35% and 13% for CD4^+^, CD8^+^ and CD19^+^ cells, respectively). In the case of CD8^+^ cells this effect was significant (Fig. 4B). However, despite this effective reduction of cell numbers both in the peripheral blood, as well as at the site of infection, we did not observe an increase in susceptibility to *S. pneumoniae* in mice rendered lymphopenic, either directly at the time of bacterial infection (Fig. 4C) or 12 h later (Fig. 4D).

**Figure 5 pone-0004840-g005:**
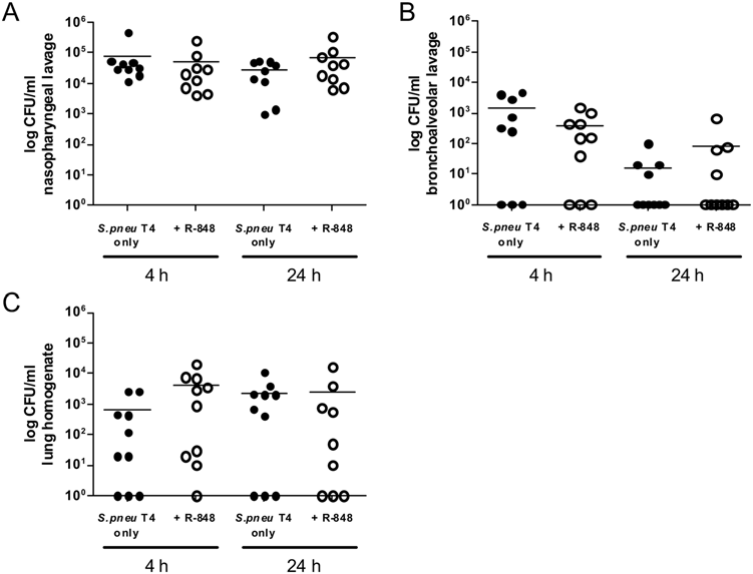
Forced lymphopenia does not interfere with bacterial clearance in *S. pneumoniae* infected hosts. C57Bl/6 mice were intranasally infected with 1×10^5^ CFU *Streptococcus pneumoniae* TIGR4 and at the same time intraperitoneally injected with PBS (•) or R-848 (○). Mice were sacrificed 4 and 24 hours later and *S. pneumoniae* CFU counts in nasopharyngeal lavage (A), bronchoalveolar lavage (B) and lung homogenates (C) were assessed. Data show values of individual mice together with group means (horizontal lines) and are compiled from two independent experiments.

Although TLR7-mediated lymphopenia did not affect the overall survival of *S. pneumoniae*-infected mice it was still possible that lymphopenia showed more subtle effects on bacterial spread. Thus we analyzed the course of bacterial deletion at three sites. As already indicated by the viability experiments, also the clearance of bacteria was unchanged in normal versus lymphopenic mice (Fig. 5).

**Figure 6:**
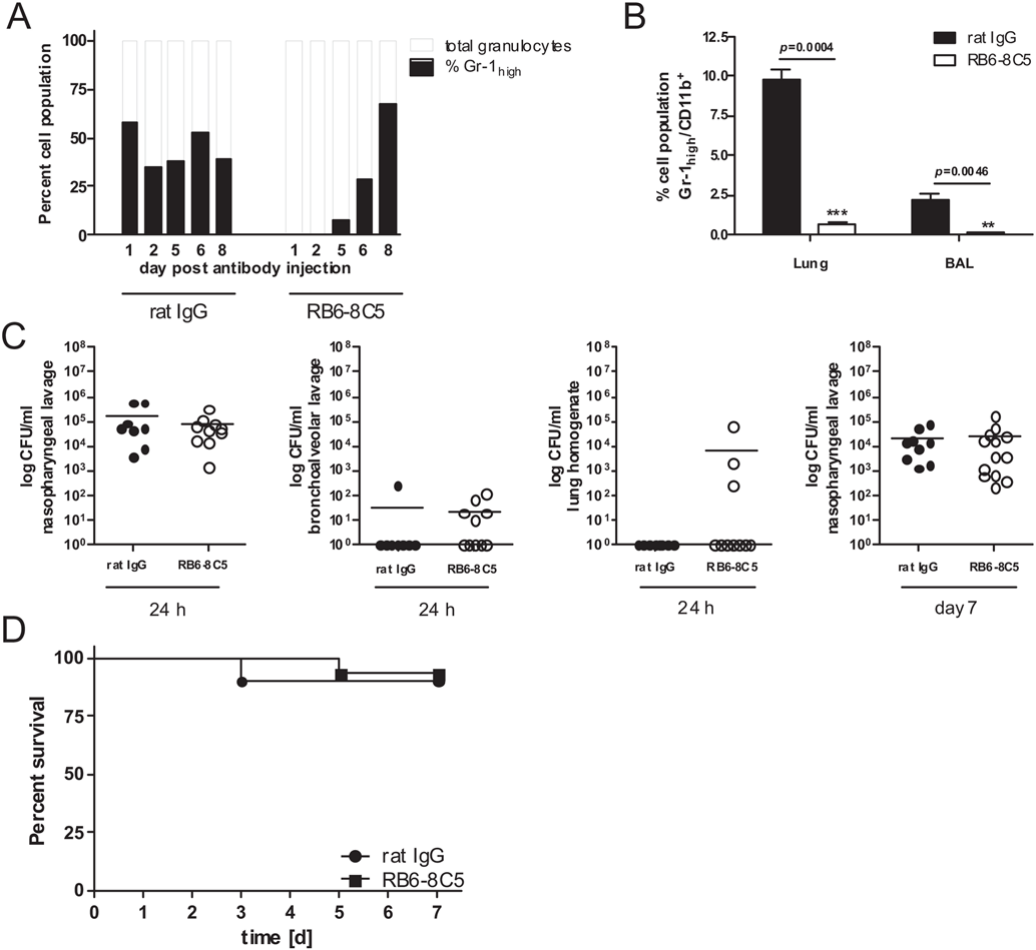
Antibody mediated depletion of PMN does not interfere with bacterial clearance in *S. pneumoniae* infected hosts. C57Bl/6 mice were intraperitoneally injected with rat IgG (•) as control or RB6-8C5 antibody (○) to deplete PMN. To confirm depletion of PMN, mouse peripheral blood samples were FACS analysed for Gr-1 positive cells on days 1, 2, 5, 6 and 7 post RB6-8C5 antibody treatment. Data show the percentage of Gr-1_high_ cells out of the respective total granulocyte population for one representative out of two analysed animals per group (A). Likewise, numbers of Gr-1_high_/CD11b^+^ cells (neutrophils) in lung tissue or BAL fluid were analysed by flow cytometry 24 h post antibody treatment (B). On the day following antibody treatment, mice were intranasally infected with 1×10^5^ CFU *Streptococcus pneumoniae* TIGR4 and sacrificed 24 hours after infection to assess *S. pneumoniae* CFU counts in nasopharyngeal lavage, bronchoalveolar lavage and lung homogenates (C). Additional groups of mice were treated equally to assess CFU counts in nasopharyngeal lavage seven days following infection (C) and survival rates (D). CFU counts show values of individual mice together with group means (horizontal lines). Data are compiled from two independent experiments with groups of at least 5 mice. For lung and BAL fluid neutrophil numbers, data from 5 mice/group are shown (+/−s.e.m.).

Finally, a critical leukocyte population that is important for early bacterial control are PMN [11], [14]. In other virus-bacteria co-infection models a reduction of PMN-numbers increases susceptibility to bacterial infection [18], [22]. While TLR7-triggering induces lymphopenia it does not equally well deplete PMN from the blood ([26] and Fig. 4A). Thus, a specific depletion of PMN might increase the susceptibility to *S. pneumoniae*-infection. We depleted PMN by injection of the monoclonal antibody RB6-8C5 specific for mouse Gr-1 [31], [32] one day before infection. As shown by repetitive measurements in individual animals a single i.p. injection of 100 µg RB6-8C5 antibody led to a very rapid (less than 15 minutes) disappearance of PMN from the peripheral blood which was almost complete and lasted at least 5 days (Fig. 6A). Importantly, the same treatment also led to an almost complete loss of PMN in lung tissue or BAL fluid, thus directly at the site of infection (Fig. 6B). Nevertheless, there was no evidence in the treated animals of an increased bacterial burden or clearance-problems at infected sites (Fig. 6C) nor did we find evidence of sepsis (data not shown). These findings were mirrored in the survival course of the experiments where transiently PMN-depleted animals did not show a significantly increased mortality-rate (Fig. 6D).

Together, these experiments showed that a forced lympho- or neutropenia did not render hosts more susceptible to superinfection with *S. pneumoniae*.

## Discussion

In this study we have investigated the role played by peripheral leukocytes in the control of a bacterial infection. Sparked by our observation that the depletion of peripheral lymphocytes by TLR7-triggering renders mice incompetent to mount a peripheral immune response [26] we reasoned that a similar mechanism might be responsible for the well known synergism of IAV and *S. pneumoniae* with IAV acting like a natural TLR7-ligand in this setting. Clearly, cellular immunity is critical for the defence against *S. pneumoniae* infection. It is well known that the lack of PMN recruitment to infected lungs can render animals susceptible for pneumococcal infection [33]–[35]. Also in other models of bacterial superinfection during ongoing viral disease, a defect of PMN has been identified as leading cause [22]. Likewise, the absence of CD4 cells increases the susceptibility to *S. pneumoniae* [16], which is a solid finding, although still difficult to explain on a mechanistic basis [14]. CD4 T cells are efficiently depleted by TLR7-ligands ([26] and Fig. 4A/B) and during IAV-infections [20]. In the light of these facts it was logical to hypothesize, that leukopenia would be a possible cause for the observed synergistic action of IAV and *S. pneumoniae*, more so, as TLR7 would provide a valuable mechanistic link.

Associating IAV-induced leukopenia with increased susceptibility to bacterial superinfection is not a novel concept [7] but still lacked a definitive experimental test. Previous studies on this issue suffered from the lack of examples where both, the time course of peripheral blood leukocyte counts and the susceptibility for bacterial superinfection were followed within the same system. Either one of the parameters was measured separately, but never both simultaneously.

In addition, the different experimental systems varied greatly e.g. in the employed pneumococcal and viral isolates as well as the strains of mice. Different mouse strains react very differently to identical treatments. PMN depletion in BALB/c mice renders them highly susceptible to pneumococcal infection [33], while we did not observe any impact of this treatment on the C57Bl/6 mice used here (Fig. 6). Genetic differences between these strains are also responsible for differences in normal pneumococcal susceptibility [36]–[38] and in this context BALB/c mice might be particularly dependent on the rapid recruitment of PMN to control early pneumococcal infiltration [36]. Another critical issue is the infectious dose of pneumococci. This varied by 5 orders of magnitude between studies from as few as 100 CFU [39] to the very high dose of 10^7^ CFU [35]. As has been demonstrated, mouse strains differ enormously in their response to low or high doses of infection [36]. It was, therefore, very difficult if not impossible to directly compare results from these different studies.

Thus it was necessary to investigate leukocyte numbers and susceptibility for synergistic function of IAV and *S. pneumoniae* in a controlled system simultaneously. We used the well established mouse adapted viral strain PR8/A/34 which is widely used for studying IAV infections in mice [39]. This was combined with the pneumococcal strain TIGR4, which is highly invasive in a mouse model and has been sequenced, thus can be considered a molecularly defined reference pathogen [27]. Our data for the first time show, that highly efficient synergistic action of IAV and pneumococcal infection can occur in the presence of normal or even increased numbers of peripheral blood leukocytes (Fig. 2). We further demonstrate that a forced reduction of peripheral leukocytes does not predispose for pneumococcal infection, at least in the model system analyzed here (Figs. 4–6).

This might point to the fact that neither PMN nor lymphocytes are necessary in the early phase of the response to this bacterial infection. Indeed, lung-resident alveolar macrophages are the first line of cellular defence for pneumococcal infections [19] and these are not depleted by the antibody RB6-8C5 [40] and most likely also not by TLR7 triggering. In addition, the lymphopenic phase after R-848-treatment is transient. Lymphocyte levels are back to normal or even higher 48 h after R-848 triggering (Fig. 4A and [26]). This time window of absence is long enough to inhibit acute responses from primed peripheral T cells [26] but obviously not sufficiently long to inhibit the protective impact of polyclonal CD4 cells on the outcome of a pneumococcal threat, which has been observed in CD4 deficient animals [14], [16]. However, PMN numbers are almost undetectable for up to 5 days after injection of RB6-8C5 (Fig. 6A), which is mirrored at the site of infection 24 h post injection, thus making a significant contribution of PMN for pneumococcal resistance in this model unlikely. Removal of PMN by a single injection of RB6-8C5 induces a profound immune suppression in C57Bl/6 mice rendering the normally resistant mice highly susceptible to a pulmonary infection with *Aspergillus fumigatus* (data not shown). But the same degree of immune suppression seems not sufficient to mediate increased susceptibility for infection with *S. pneumoniae*.

Our study now firmly establishes, that normal numbers of circulating leukocytes do not protect from a lethal bacterial superinfection but that the lack of peripheral leukocytes alone does not predispose for the infection. What might be the reason for this phenomenon? Recent evidence suggests, that Interferon-γ produced massively by the immune system during viral defence renders alveolar macrophages unable to phagocytose incoming bacteria [19]. However, a previous paper from the same group finds, that Interferon-γ-mediated recruitment of PMN to the lung is a protective mechanism for pneumococcal infection [33], calling the importance of the other finding for pneumococcal susceptibility into question. Nevertheless, these results belong to a group of observations that link the natural antiviral response with a toxic impact on endogenous immune cells like alveolar macrophages [19] or PMN [18], [22]. A different line of findings demonstrates that an overt immune response, mainly of PMN origin, is responsible for a pathologic destruction of lung tissue in the attempt to fight the bacterial superinfection [17], [39]. This has recently been linked to the IAV-protein PB1-F2 with the 1918-strain being the source of a particularly virulent version of this protein [39]. However, IAV-1918 is special leading to an uncontrolled and not well timed cytokine storm in infected animals [41] making this human-adapted strain also highly pathogenic for mice [42], [43].

However, these findings can only partially explain the processes in co-infected animals in the model used here. We have used the viral strain PR8/A/34 leading to a relatively mild form of the viral infection (Fig. 1). The majority of mice can cope well with the infection and clear it without a large health burden which is in sharp contrast to IAV-1918. What causes increased superinfection here must remain open. Our data show that depletion of peripheral leukocytes is not critical (Fig. 2, 4–6). In contrast, analyses of BAL from co-infected mice in our model suggest a highly increased infiltration of the lung with PBMC (data not shown). Unable to protect from superinfection, these cells could even be toxic and their depletion might result in a health benefit in a mouse model of pneumococcal infection [44]. If this can be further substantiated, it might be considered for future IAV epidemics to combine a prophylactic antibiotic treatment, which on its own is not protective [45] with some form of medical control for overt leukocyte recruitment and the uncontrolled release of pro-inflammatory cytokines.
